# Seeing is believing: what is on the surface of silver nanocrystals suspended in their original reaction solution†

**DOI:** 10.1039/d4sc00730a

**Published:** 2024-04-01

**Authors:** Qijia Huang, Dong Qin, Younan Xia

**Affiliations:** a School of Chemistry and Biochemistry, Georgia Institute of Technology Atlanta Georgia 30332 USA younan.xia@bme.gatech.edu; b School of Materials Science and Engineering, Georgia Institute of Technology Atlanta Georgia 30332 USA; c The Wallace H. Coulter Department of Biomedical Engineering Georgia Institute of Technology and Emory University Atlanta Georgia 30332 USA

## Abstract

Colloidal synthesis of inorganic nanocrystals always involves a multitude of ionic and molecular species. How the chemical species affect the evolution of nanocrystals remains a black box. As an essential ingredient in the polyol synthesis of Ag nanocubes, Cl^−^ has been proposed to co-adsorb on the surface with poly(vinyl pyrrolidone) (PVP) to facilitate shape evolution. However, there is still no direct evidence to confirm the presence of Cl^−^ on the surface of Ag nanocubes while they are suspended in the original reaction solution. By leveraging the high sensitivity of surface-enhanced Raman scattering, here we offer direct evidence, for the first time, by resolving the Ag–Cl vibrational peak at 240 cm^−1^. This characteristic peak disappears if the synthesis is conducted in the absence of Cl^−^. Instead, three peaks associated with CF_3_COO^−^ (from the precursor to Ag) are observed. When the sample is diluted with ethylene glycol, all the peaks associated with CF_3_COO^−^ decrease proportionally in intensity, implying the involvement of chemisorption and negligible desorption during dilution. The chemisorbed CF_3_COO^−^ is readily replaced by Cl^−^ due to their major difference in binding strength. The co-adsorbed Cl^−^ forces the carbonyl group of PVP binding to the Ag surface to take a more perpendicular configuration, enhancing its peak intensity. Altogether, these findings shed new light on the roles played by various chemical species in a successful synthesis of Ag nanocubes.

## Abstract 2

Surface-enhanced Raman scattering was used to resolve the chemical species, including chloride ions, on the surface of Ag nanocrystals in their original reaction solution, avoiding changes to the surface while eliminating possible artifacts.

## Graphical abstract

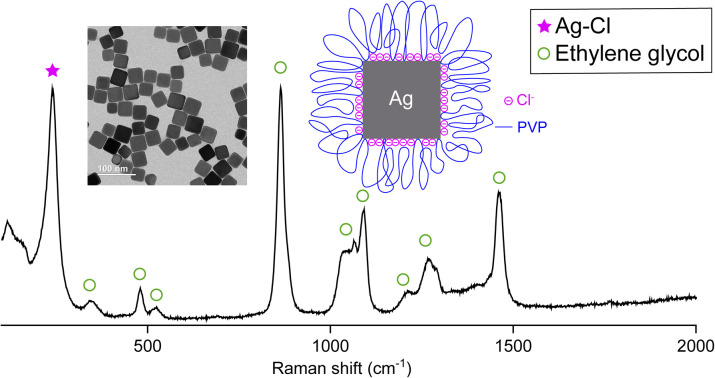

## 1 Introduction

Inorganic nanocrystals synthesized using colloidal methods have found widespread use in a broad range of applications. As a notable example, Ag nanocubes have been extensively studied owing to their fascinating properties related to localized surface plasmon resonance (LSPR) and surface-enhanced Raman scattering (SERS),^1–8^ as well as their applications as an antimicrobial agent^9,10^ and a catalyst toward the epoxidation reaction.^11,12^ Many colloidal methods have been developed for the synthesis of Ag nanocubes with uniform and controllable sizes, with the most successful ones built around polyol reduction.^13–22^ In a typical polyol synthesis, a Ag(i) precursor such as CF_3_COOAg or AgNO_3_ is reduced by ethylene glycol (EG) or diethylene glycol (DEG) at an elevated temperature in the presence of PVP.^14,18,19^ A variety of ionic species such as Cl^−^ and SH^−^ are also added to help improve the robustness and reproducibility of the synthesis by controlling both the nucleation and growth mechanisms.^23,24^ In addition to its role as a coordination ligand for oxidative etching and thus selective removal of the twinned seeds in the nucleation step, recent studies involving both theoretical and experimental efforts proposed the possible co-adsorption of Cl^−^ with PVP on Ag{100} facets to induce and direct the evolution of a cubic shape.^25^ However, there is still no direct evidence to support the presence of Cl^−^ on the surface of Ag nanocubes while they are suspended in the original reaction solution.

The current evidence that supports the adsorption of Cl^−^ on the surface of Ag nanocubes mainly comes from characterization techniques such as X-ray photoelectron spectroscopy (XPS) and energy-dispersive X-ray (EDX) mapping.^26–28^ Both analyses can only be conducted with dried samples placed in a high vacuum. The extra step of sample preparation may introduce uncertainties or artifacts, and even alter the surface composition. For example, the Cl^−^ ions may stay free in the reaction solution and be forced to adsorb onto the surface during the drying process. Exposure of a dried sample of Ag nanocubes to air may also cause the formation of oxides and/or sulfides,^29,30^ making it difficult to elucidate the native composition of the surface. Ideally, one should keep Ag nanocubes in their original reaction solution while collecting information from their surface only. A recent study involving electrochemical measurements attempted to address this issue but such measurements only apply to single-crystal substrates rather than nanocrystals prepared using a colloidal method.^25^ In contrast, surface-enhanced Raman scattering (SERS) easily stands out as a viable technique for its unique capability to elucidate the molecular species adsorbed on the surface of nanocrystals made of plasmonic metals such as Ag, Au, and Cu with high sensitivity.^31–34^ With a higher resolution than infrared spectroscopy (IR) in the low-wavenumber region, SERS provides comprehensive information on the vibrational “fingerprint” of a chemical species in proximity to the surface of plasmonic nanocrystals.^35,36^ As another advantage, SERS can also be conducted with liquid samples to enable *in situ* characterizations without involving complications from any extra step of sample preparation.

Herein, we report the use of SERS to analyze the native surface of Ag nanocubes suspended in the original reaction solution of a polyol synthesis. By comparing the SERS spectra recorded from samples prepared in the presence and absence of Cl^−^, we confirm the presence of Cl^−^ on the surface of Ag nanocubes suspended in the original reaction solution by unambiguously resolving the Ag–Cl vibrational peak at 240 cm^−1^. We also elucidate the presence of other surface-adsorbed species, including CF_3_COO^−^ (from the precursor) and PVP (the colloidal stabilizer and surface capping agent). It is found that CF_3_COO^−^ can only adsorb on Ag surface in the absence of Cl^−^ and the pre-adsorbed CF_3_COO^−^ can be readily replaced by Cl^−^ due to their major difference in binding strength. Indeed, Cl^−^ and PVP can co-adsorb on Ag surface and the presence of Cl^−^ can force the carbonyl group of PVP to take a more perpendicular configuration and thereby enhance the SERS intensity of the carbonyl group.

## 2 Results and discussion

**Fig. 1:**
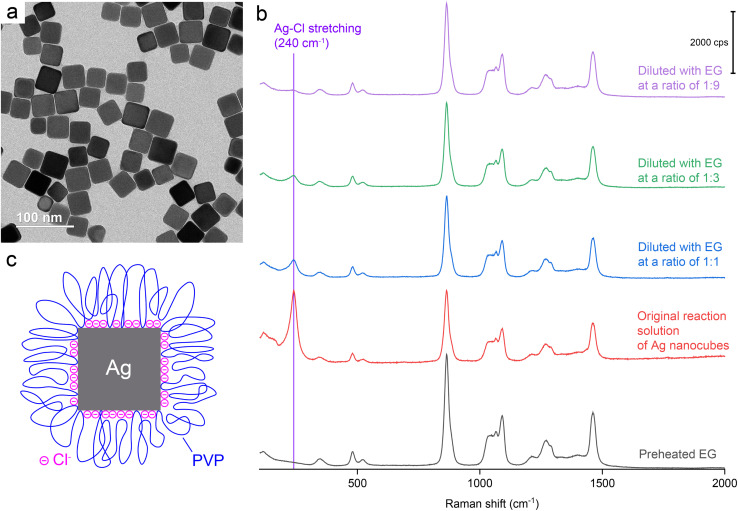
(a) A typical TEM image of the Ag nanocubes. (b) Raman spectrum of EG (preheated at 150 °C for 1 h) and SERS spectra recorded from the original reaction solution of Ag nanocubes and after it had been diluted with preheated EG at various ratios. (c) A model detailing the native surface of a Ag nanocube suspended in the original reaction solution.

We started our SERS measurements with Ag nanocubes. By following a published protocol,^18^ we synthesized Ag nanocubes with an average edge length of 35.8 ± 3.2 nm. The reaction was quenched by immersing the flask in an ice bath. A small portion of the reaction solution was mixed with acetone to crush out the nanocubes for characterization by transmission electron microscopy (TEM). A typical TEM image is shown in Fig. 1a. The remaining solution was directly used for SERS measurements in an effort to preserve the pristine surface of the nanocubes in the original reaction solution. The same reaction solution was also diluted with ethylene glycol (EG) at various ratios. The EG used for all dilution experiments was preheated at 150 °C for 1 h to remove water and thus ensure consistency with the EG used for the synthesis of nanocubes. Fig. 1b shows a Raman spectrum of the preheated EG and the SERS spectra recorded from the original reaction solution before and after dilution with EG. Before dilution, the SERS spectrum shows a strong peak at 240 cm^−1^, which can be assigned to the stretching mode of Ag–Cl solid (*ν*_Ag–Cl_).^37^ This peak was the only spectral feature that differentiated the SERS spectra of the reaction solutions from the Raman spectrum of pure EG. All the other peaks could be assigned to EG.^38^

To gain a more accurate understanding of the surface of Ag nanocubes during their polyol synthesis, we conducted a SERS measurement with the original reaction solution held at a temperature close to what (about 150 °C) was used for the synthesis. In this case, the hot solution was directly transferred from the reaction container to a sample holder supported on a heating pad for the SERS measurement. As shown in Fig. S1,† a peak corresponding to the *ν*_Ag–Cl_ mode was still resolved at 238 cm^−1^. Compared to the spectrum recorded at room temperature, the *ν*_Ag–Cl_ peak observed at an elevated temperature of about 150 °C slightly shifted to a lower frequency while showing a weakened intensity relative to the SERS peaks of EG. The slight redshift at the increased temperature reflects a minor increase in the Ag–Cl bond length and thus weakened bond strength because of thermal expansion. The decrease in intensity suggested the desorption of Cl^−^ from the surface of Ag nanocubes due to the weakened interaction between them. This SERS data clearly confirms the presence of Cl^−^ on the surface of Ag nanocubes during a polyol synthesis despite its reduced coverage density at an elevated temperature.

It is worth noting that the *ν*_C=O_ peak of PVP was essentially invisible in the SERS spectra recorded from all the samples without/with EG dilution, indicating that the SERS cross-section of *ν*_C=O(Ag)_ was much smaller than that of *ν*_Ag–Cl_ and/or the number of C=O bonds in the probed volume was much lower than that of Ag–Cl bonds. The absence of *ν*_C=O_ peak can also be attributed to other factors: (i) the presence of Cl^−^ on the surface limited the number of C=O groups directly bonding to the surface and thus reducing the intensity of *ν*_C=O_; (ii) the strong solvation by EG caused the free C=O groups of the adsorbed PVP to stay away from the surface of Ag nanocubes, further reducing the intensity of *ν*_C=O_; and (iii) the original reaction solution contained Ag nanocubes at a lower concentration (3.4 × 10^11^*vs.* 7.2 × 10^11^ particles per mL) relative to what was used in a prior study.^39^ Based on the SERS data, a model can be proposed to account for the chemical species present on the surface of Ag nanocubes when suspended in the original reaction solution (see Fig. 1c). This model differs from what has been reported in literature in two aspects: (i) the inclusion of a dense layer of Cl^−^ chemisorbed on the surface and (ii) the reduced density of C=O groups directly binding to the Ag surface and thus larger loops taken by the PVP chains.^39,40^ The as-prepared Ag nanocubes in an aqueous suspension had a zeta potential of −34.4 mV, confirming the presence of a dense layer of chemisorbed Cl^−^ on the surface. In reality, the highly-solvated and extended PVP chains tended to intertwine with each other to form a three-dimensional network, making it very difficult to precipitate out the particles by centrifugation only.

**Fig. 2:**
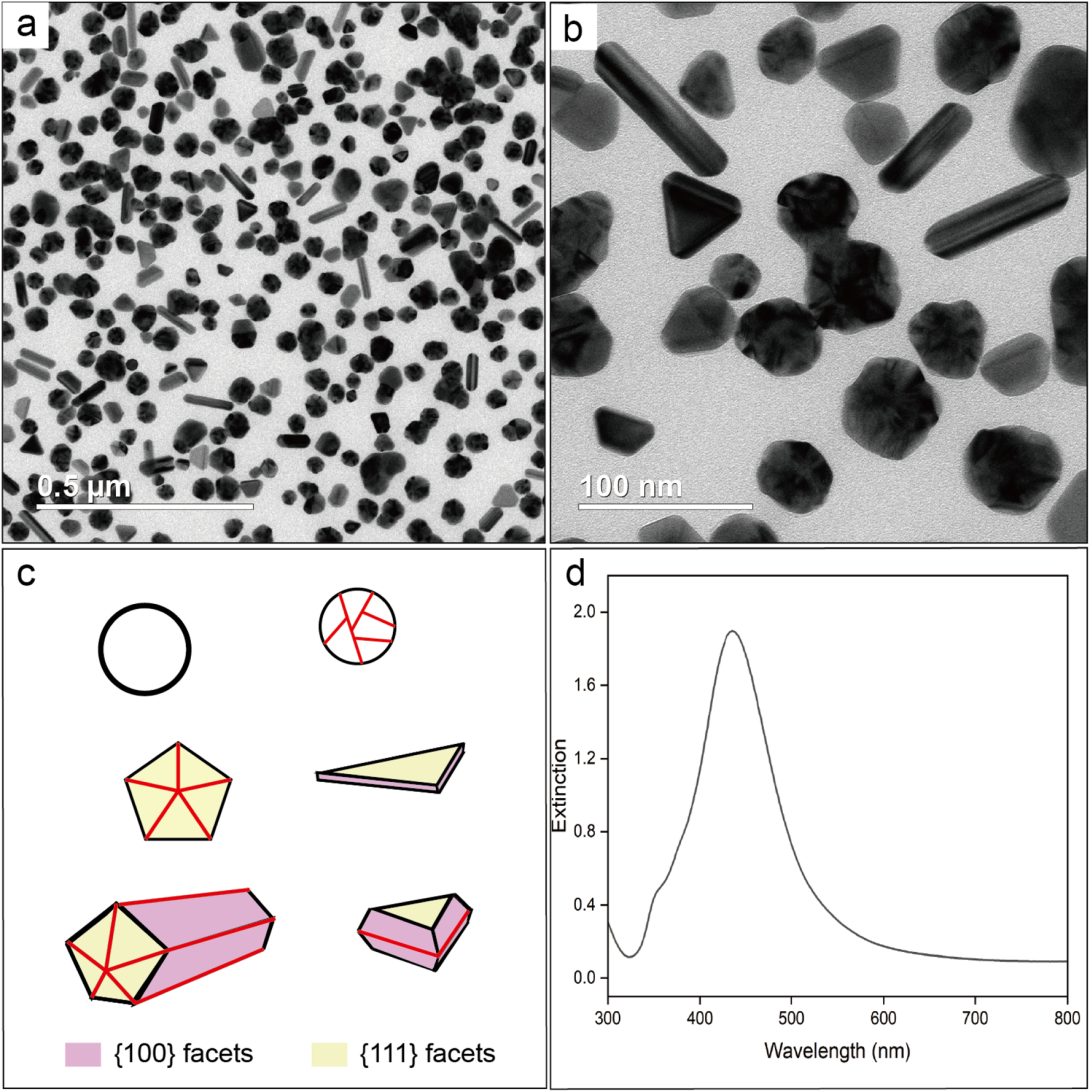
(a and b) TEM images of the Ag irregular particles prepared in the absence of Cl^−^, which were collected by precipitation with acetone, followed by centrifugation and washing with water. (c) Models showing the typical shapes (with the facets color coded) taken by the nanocrystals. The twin boundaries are marked by red lines. (d) UV-vis spectrum recorded from a suspension of the Ag nanocrystals in the original reaction solution.

If the oxygen atom of the C=O group in PVP coordinates with the Ag surface, it can result in Ag–O stretching mode with a characteristic Raman shift in the range of 220–240 cm^−1^.^41,42^ To validate that the SERS peak observed at 240 cm^−1^ arises from Ag–Cl rather than Ag–O stretching mode, we conducted a control experiment by voiding HCl from the protocol used for the synthesis of Ag nanocubes while keeping all other parameters the same. Fig. 2a and b, shows TEM images of the sample prepared in the absence of Cl^−^. Most of the particles showed irregular shapes due to the dominance of random twinning (77.4%), with a small portion of them taking the forms of rods (9.7%), triangular plates (3.1%), truncated bipyramids (8.8%), and single-crystal nanospheres (1.0%). As such, the surface of these nanocrystals was enclosed by a mix of {111} and {100} facets, in contrast to the dominance of {100} facets on the nanocubes. This outcome is consistent with what has been reported in literature.^18^ Models of the typical shapes are shown in Fig. 2c, with the twin boundaries marked by red lines. Fig. 2d shows a UV-vis spectrum taken from the reaction solution after dilution with the preheated EG. No shoulder peak was found at wavelengths beyond the major LSPR peak, indicating that the as-prepared particles were well dispersed in EG without forming aggregates even during the dilution with EG. In this case, the PVP could still serve as an effective colloidal stabilizer by adsorbing onto the surface of the particles in the absence of Cl^−^. The as-prepared Ag particles in an aqueous suspension had a zeta potential of −12.1 mV. This value was less negative than that of the nanocubes, suggesting that the surface charges on the nanocubes were mainly contributed by Cl^−^. In addition, we measured the hydrodynamic diameters of the nanocubes and the nanocrystals in EG using dynamic light scattering. As shown in Fig. S2,† the average size of the nanocubes revealed by TEM was smaller than that of the irregular particles. However, the hydrodynamic diameter of the nanocubes was found to be 81.9 ± 1.4 nm, much greater than that (48.6 ± 0.6 nm) of the irregular particles. This trend indicates that a dense layer of Cl^−^ on the surface of the nanocubes resulted in the formation of bigger loops for the adsorbed PVP when compared with the irregular particles prepared in the absence of Cl^−^.

**Fig. 3:**
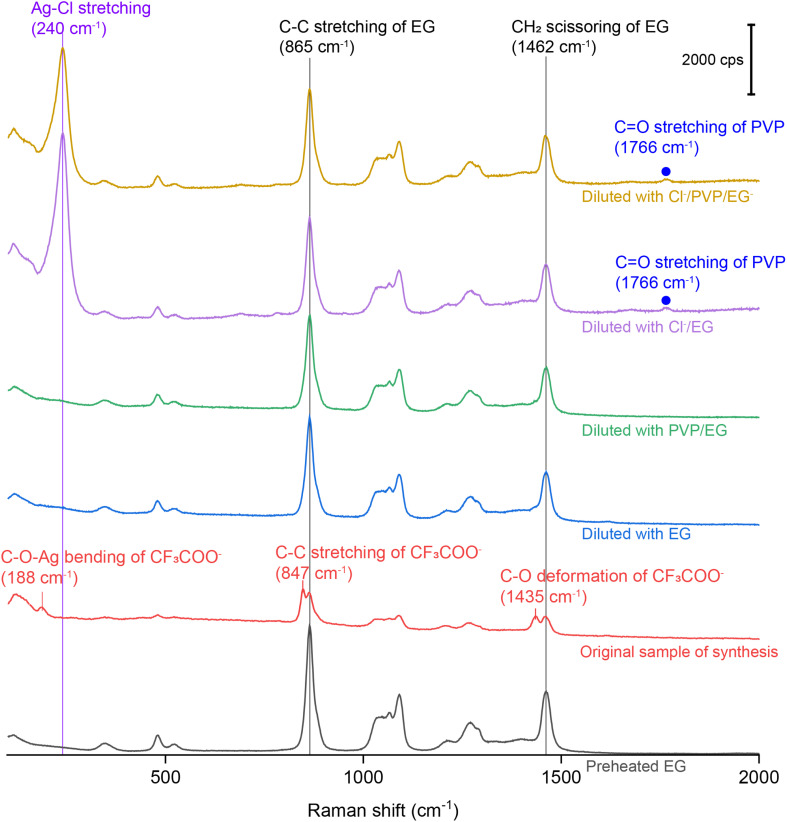
Raman spectrum of the preheated EG, SERS spectra of the as-obtained Ag irregular particles in the original reaction solution containing no Cl^−^ and after it had been diluted by 10 times with EG, EG containing PVP, EG containing Cl^−^, and EG containing both PVP and Cl^−^.

For SERS measurements, we also quenched the reaction by immersing the flask in an ice bath and then diluted by a factor of 10 with EG, EG containing PVP, EG containing Cl^−^, and EG containing both PVP and Cl^−^, respectively. The EG used for dilution was also preheated at 150 °C for 1 h whereas the final concentrations of PVP and Cl^−^ were kept the same as those used in the synthesis of Ag nanocubes. Fig. 3 shows the Raman and SERS spectra recorded, respectively, from pure EG and the original solution for the synthesis of Ag irregular particles in the absence of Cl^−^. As expected, we did not observe any peak around 240 cm^−1^, confirming that this peak originated from Ag–Cl rather than Ag–O stretching mode. A new peak was observed at 188 cm^−1^, which could be assigned to the bending mode of C–O–Ag (*δ*_C–O–Ag_) due to the adsorption of CF_3_COO^−^ on the surface of the nanoparticles. Because CF_3_COO^−^ did not compete favorably with Cl^−^ for surface adsorption, this peak was missing in the SERS spectrum of the nanocubes (Fig. 1). Interestingly, compared with the Raman spectrum of EG, two additional new peaks appeared at 847 and 1435 cm^−1^ and they could be assigned to the C–C stretching (*ν*_C–C_) and C–O deformation modes (*δ*_C–O_) of CF_3_COO^−^, respectively.^43^ Again, these two peaks were missing in the SERS spectrum of the nanocubes, suggesting that CF_3_COO^−^ could not adsorb onto the Ag surface in the presence of a stronger adsorbate such as Cl^−^. When the original sample of Ag nanocrystals was diluted by 10 times with EG, the SERS peaks at 188, 847 and 1435 cm^−1^ all disappeared. We also noticed that the spectrum of the original sample diluted with EG containing PVP was essentially identical to that involving EG only. The disappearance of these three peaks could be attributed to the decrease in particle concentration caused by dilution, leading to a proportional decrease in SERS peak intensity, in addition to the desorption expected for a weakly-bound adsorbate. With the addition of Cl^−^ into the EG used for dilution, we were able to resolve the peak at 240 cm^−1^, further supporting the assignment of this peak to the Ag–Cl stretching mode. It is worth noting that a small peak positioned at 1766 cm^−1^, with its assignment to the *ν*_C=O_ of PVP adsorbed on the surface of Ag nanocubes suspended in EG, was observed in both cases involving Cl^−^ for dilution. The peak position is consistent with what was reported in our previous study.^39^ This observation suggests that Cl^−^ could compete favorably with PVP for surface adsorption and enhance the SERS intensity of *ν*_C=O_ corresponding to the PVP co-adsorbed on the surface. Interestingly, this peak was missing in the SERS spectrum recorded from the Ag nanocubes (Fig. 1). The difference can be explained by assuming that the adsorption of Cl^−^ had a preference toward the {100} facets on the Ag nanocubes relative to the {111} facets on the Ag nanoparticles.^25^ As such, the amount of PVP capable of anchoring to the surface of the nanocubes would be significantly reduced relative to that on the irregular particles, leading to a weaker SERS peak intensity.

The peak assignment for CF_3_COO^−^ was also validated by replacing CF_3_COOAg with AgNO_3_ as a precursor to synthesize Ag nanocrystals in the absence of Cl^−^. As shown by the TEM images in Fig. S3,† the as-obtained particles had a similar size distribution and UV spectrum as those shown in Fig. 2, suggesting comparable SERS activity for these two different samples. Fig. S4† shows the SERS spectra recorded from the original and EG-diluted samples of the Ag irregular particles prepared with AgNO_3_ in the absence of Cl^−^. As expected, the SERS peaks at 847 and 1435 cm^−1^ disappeared from the spectra, confirming the assignment of these two peaks to the vibrations of CF_3_COO^−^. Additionally, compared with the SERS spectrum of Ag nanoparticles prepared with CF_3_COOAg in Fig. 3, the peak at 188 cm^−1^ also disappeared, further confirming the assignment of this peak to the bending mode of C–O–Ag. According to literature, the Raman peak associated with the bending mode of C–O–Ag in CH_3_COOAg was located at 224 cm^−1^.^44^ The electron-withdrawing nature of the –CF_3_ group in CF_3_COO^−^ can weaken the O–Ag bond, causing the *δ*_C–O–Ag_ band to red-shift to 188 cm^−1^.

**Fig. 4:**
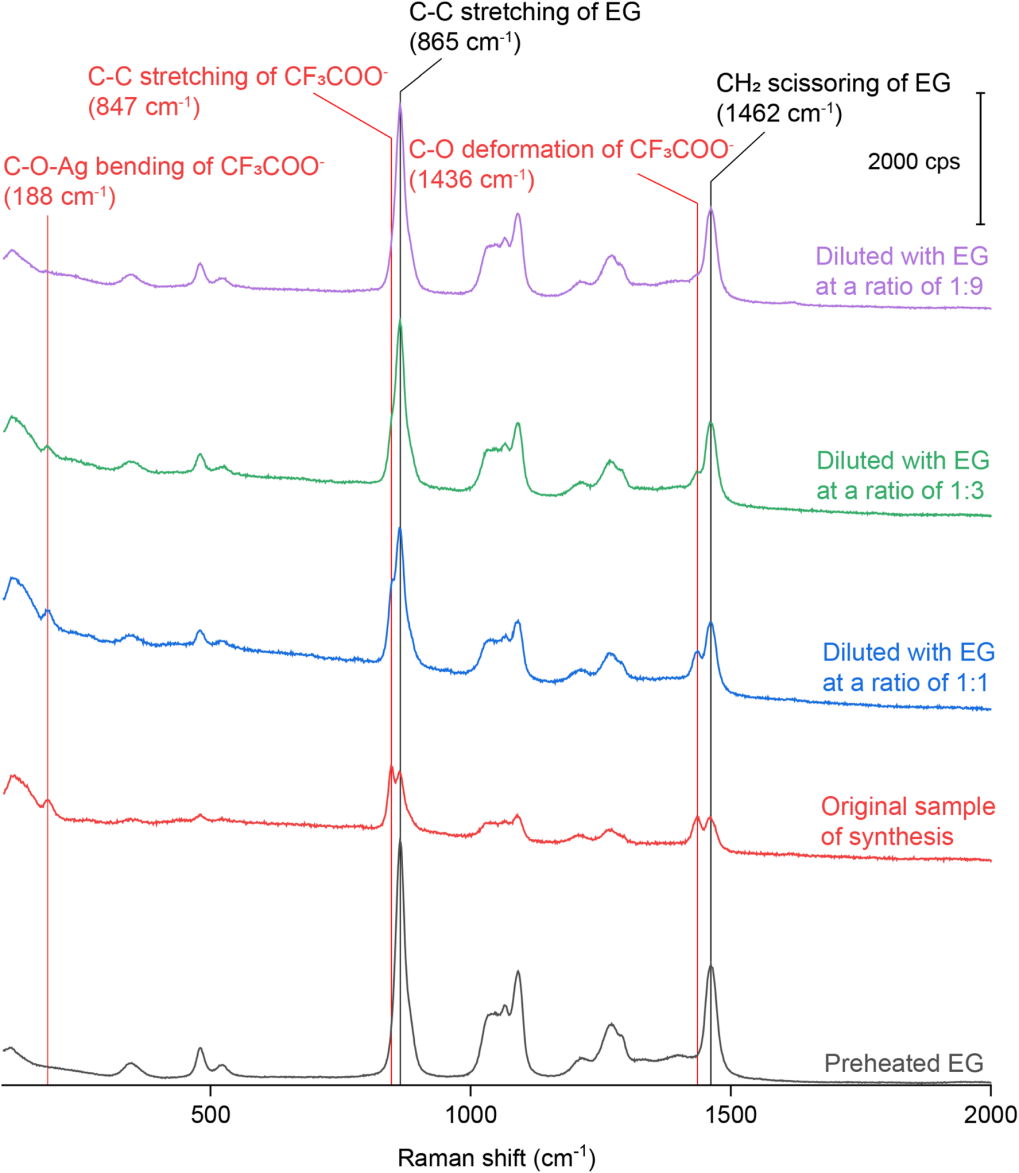
Raman spectrum of preheated EG and SERS spectra recorded from the original reaction solution of Ag irregular particles synthesized using CF_3_COOAg and after it had been diluted with the EG at various ratios.

We also diluted the original reaction solution containing Ag irregular particles (prepared using CF_3_COOAg) with pure EG at various ratios. Fig. 4 shows the Raman spectrum of pure EG and the SERS spectra collected from the original and diluted samples. As the dilution factor was increased, the areas of both the peaks at 864 and 1462 cm^−1^ increased accordingly. These two peaks belong to the Raman shifts of the C–C stretching and CH_2_ scissoring bands of EG. It is worth mentioning that when we collected SERS spectra from a liquid sample, we initially focused on the surface of the coverslip and then tuned the knob by a certain degree to move the focal point into the liquid. As such, the probed volume should be well within the sample. In this case, the higher concentration of Ag nanoparticles would cause more attenuation of light between the coverslip and the probed volume, reducing the intensities of the Raman (from EG) and SERS signals. As the concentration of Ag nanoparticles was reduced, the extent of light attenuation would decrease to give a stronger Raman signal for the EG within the probed volume. When the dilution factor was increased, we noticed that the ratio between the areas of the peaks at 847 and 864 cm^−1^ decreased significantly, as well as the ratio between the areas of the peaks at 1435 and 1462 cm^−1^, as shown in Fig. S5.† If we take the intensity of the EG peak at 1462 cm^−1^ as a reference, the ratio between the areas of the peaks at 1435 cm^−1^ (deformation mode of C–O in CF_3_COO^−^) and 1462 cm^−1^ (scissoring mode of CH_2_ in EG) decreased proportionally as the dilution factor was increased, indicating that the CF_3_COO^−^ detected by SERS chemisorbed on the surface of the Ag nanoparticles during synthesis. Interestingly, we found that the ratio between the areas of the peaks at 847 cm^−1^ (*ν*_C–C_ of CF_3_COO^−^) and 865 cm^−1^ (*ν*_C–C_ of EG) decreased at a faster rate than the increase in dilution factor.

**Fig. 5:**
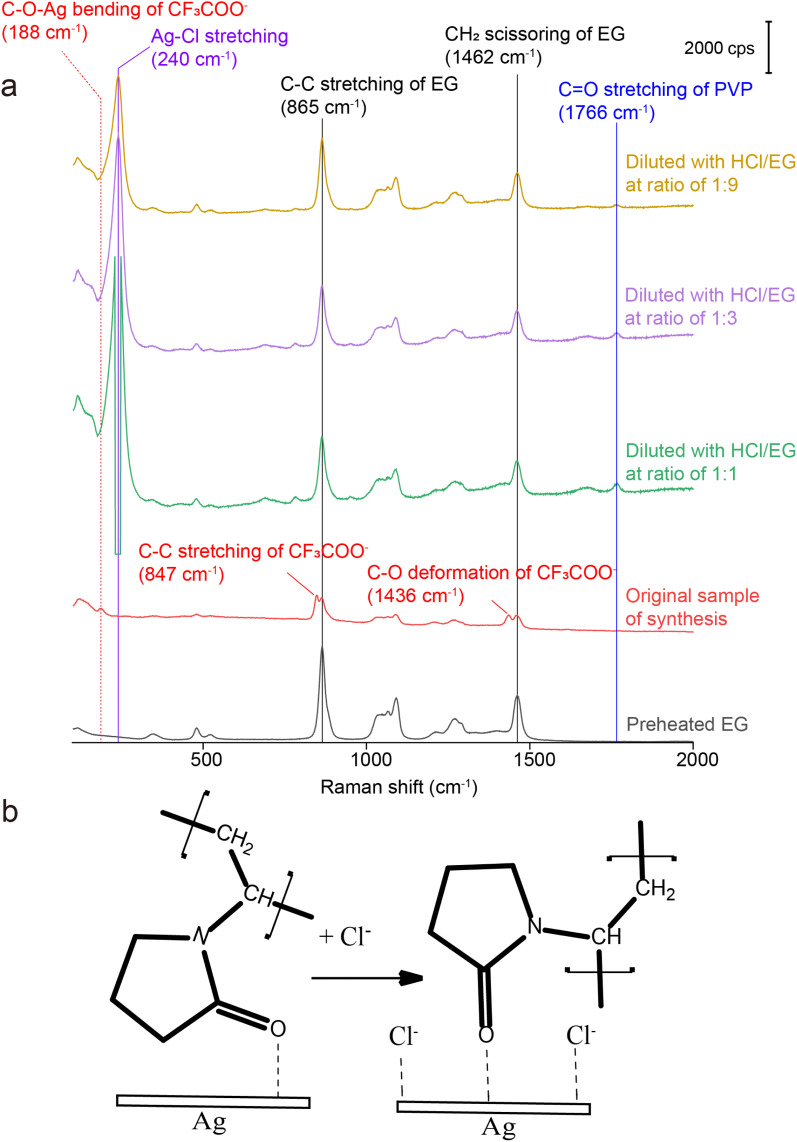
(a) Raman spectrum of preheated EG and SERS spectra recorded from the original reaction solution of Ag irregular particles synthesized using CF_3_COOAg and after it had been diluted with HCl/EG at various ratios, with the final concentration of Cl^−^ being 0.21 mM; and (b) schematic showing the effect of chemisorbed Cl^−^ on the orientation of C=O group of PVP relative to the Ag surface.

To better understand the roles of PVP and Cl^−^, we also collected SERS spectra from the original reaction solution of the Ag irregular particles prepared with CF_3_COOAg after dilution with EG containing PVP and HCl, respectively. The final concentrations of PVP and HCl in the diluted samples were kept the same as those in the reaction solution used for the synthesis of Ag nanocubes. The SERS spectra shown in Fig. S6† are nearly identical to those in Fig. 4. Based on this observation, we argue that the number of PVP chains per unit area anchored to the surface of Ag irregular particles remained the same when varying the concentration of PVP. When the original reaction solution was diluted with HCl/EG, we observed a strong peak at 240 cm^−1^, further confirming the assignment of this peak to *ν*_Ag–Cl_ (the peak intensity became saturated when the dilution factor was 1 : 1). Compared with the spectra in Fig. 4 and S6,† it was difficult to resolve the peak at either 188, 847, or 1436 cm^−1^ in Fig. 5a after introducing Cl^−^, even at low dilution factors. These three peaks corresponded to *δ*_C–O–Ag_, *ν*_C–C_, and *δ*_C–O_ of CF_3_COO^−^, respectively, indicating the absence of CF_3_COO^−^ from the surface of Ag irregular particles due to the much stronger binding of Cl^−^ than CF_3_COO^−^ and thus ligand replacement. Interestingly, we also observed a peak at 1766 cm^−1^, which could be assigned to the C=O stretching mode of PVP, *ν*_C=O_.^39^ We argue that the presence of Cl^−^ can probably vary the contact angle between the Ag surface and the C=O group of PVP, resulting in a more perpendicular orientation and thus stronger SERS signal, as illustrated in Fig. 5b. In the absence of Cl^−^, the C=O group of PVP might adopt a smaller tilting angle with the Ag surface,^42^ weakening the SERS peak intensity for the C=O stretching mode.^45,46^ After the introduction of HCl, the Cl^−^ could chemisorbed on the surface of the irregular particles to promote perpendicular orientation as a result of steric hindrance and/or electrostatic repulsion.

## 3 Conclusion

In summary, we have demonstrated the use of SERS as a powerful tool to elucidate the chemical species adsorbed on the surface of Ag nanocrystals while they are still dispersed in the original reaction solution. Specifically, we provide direct evidence to confirm the presence of Cl^−^ on the surface of Ag nanocubes synthesized using a HCl-mediated polyol method. When the synthesis is conducted in the absence of Cl^−^, we observe the chemisorption of CF_3_COO^−^ from the precursor, but this species can be readily replaced by the newly added Cl^−^, indicating a stronger interaction between Cl^−^ and Ag surface. Our results suggest that Cl^−^, in addition to its role as a coordination ligand for the selective removal of twinned seeds by oxidative etching, also plays a pivotal role in directing the evolution of a cubic shape *via* co-adsorption with PVP. The co-adsorbed Cl^−^ pushes the surface-bound carbonyl group of PVP into a more perpendicular configuration, enhancing the peak intensity of *ν*_C=O_–_Ag_. Collectively, this work not only sheds new light on the intricate molecular interactions occurring on the surface of Ag nanoparticles during their polyol synthesis, but also offers a viable strategy to enhance the SERS intensity by manipulating the binding configuration of the probe molecule relative to the Ag surface.

## Data availability

All relevant data has been included in the manuscript and ESI.†

## Author contributions

Q. H. and D. Q. conceived the project. Q. H. conducted the experiments. Q. H., D. Q, and Y. X. analyzed the data. Q. H. wrote the original manuscript. D. Q. and Y. X. reviewed and revised the manuscript.

## Conflicts of interest

There are no conflicts to declare.

## Supplementary Material

SC-015-D4SC00730A-s001
