# Supplementary material for: Seeing is believing: what is on the surface of silver nanocrystals suspended in their original reaction solution
Source: Chem Sci. 2024 Apr 1;15(17):6321–30. doi: 10.1039/d4sc00730a (PMC11062097; doi:10.1039/d4sc00730a)
Supplement: SC-015-D4SC00730A-s001 [file SC-015-D4SC00730A-s001.pdf]

Electronic Supplementary Material (ESI) for Chemical Science.
This journal is © The Royal Society of Chemistry 2024

Supporting Information

Seeing is believing: what is on the surface of silver nanocrystals suspended in their original reaction solution

Qijia Huang,[a] Dong Qin,[b] and Younan Xia,[a, c,*]

[a]School of Chemistry and Biochemistry, Georgia Institute of Technology, Atlanta, Georgia 30332, United States

[b]School of Materials Science and Engineering, Georgia Institute of Technology, Atlanta, Georgia 30332, United States

[c]The Wallace H. Coulter Department of Biomedical Engineering Georgia Institute of Technology and Emory University, Atlanta, Georgia 30332, USA

Table of the contents

1. Experimental Section 1
2. Supplementary figures 4

1. Experimental Section

Chemicals and Materials. Poly(vinylpyrrolidone) (PVP) with an average molecular weight of 55000 (PVP-55k), silver trifluoroacetate (CF_3COOAg, ≥99.99%, trace metal basis), sodium hydrosulfide hydrate ($NaHS \cdot xH_2O$), and aqueous hydrochloric acid (HCl, 37%) were all purchased from Sigma-Aldrich. Ethylene glycol ($HOCH_2CH_2OH$, EG) was ordered from J. T. Baker. Acetone (HPLC grade, 99.5+%) was obtained from Alfa Aesar. All the chemicals were used as received. Deionized (DI) water with a resistivity of 18.2 MΩ·cm at room temperature was used

throughout experiments.

Synthesis of Ag Nanocubes. The Ag nanocubes were synthesized by stepwise introducing 0.24 mL of NaSH (3 mM in EG), 2.0 mL of HCl (3 mM in EG), 5.0 mL of PVP-55k (20 mg/mL in EG), and 1.6 mL of CF_3COOAg (282 mM in EG) into 20 mL of preheated EG (hosted in a 100-mL flask) after its temperature had reached 150 °C. After the introduction of CF_3COOAg, we monitored the main LSPR peak position of the reaction mixture using a UV-vis spectrometer. Specifically, a few drops of the reaction solution were withdrawn from the flask using a glass pipet and diluted with water in a cuvette, followed by the collection of its extinction spectrum. When the main LSPR peak reached 430 nm, the reaction was immediately quenched by immersing the flask in an ice bath. A small amount of the solid product was crushed out using acetone, washed with water twice, and then dispersed in water for TEM sample preparation. A more detailed description of the protocol can be found in a prior publication.

Synthesis of Ag Irregular Particles with CF_3COOAg as a Precursor in the Absence of Cl^-. The Ag irregular particles were synthesized in the absence of Cl^- by following the exact protocol used for the synthesis of Ag nanocubes except that no HCl was introduced.

Synthesis of Ag Irregular Particles with $AgNO_3$ as a Precursor in the Absence of Cl^-. The Ag irregular particles were synthesized by following the exact protocol used for the synthesis of Ag nanocubes except that CF_3COOAg was replaced with the same molar amount of $AgNO_3$ while no HCl was added.

Raman and SERS Measurements. An aliquot of 25 μL was withdrawn from the reaction solution for the synthesis of Ag nanoparticles after it had been cooled down to room temperature and applied to SERS measurement. In a typical process, we transferred the aliquot into a cell made of poly(dimethyl siloxane) (PDMS), covered with a glass coverslip, and placed on the sample stage of the microscope. We then collected SERS spectra in the extended mode at an excitation wavelength of 532 nm, together with a 100× objective lens, a laser power at 25 mW, and a collection time of 10 s. The reaction solution was also diluted with EG at different ratios. The EG was preheated at 150 °C for 1 h and then cooled down to room temperature prior to the dilution. An aliquot of 25 μL was withdrawn from the preheated EG or each of the diluted samples for Raman and SERS measurements, respectively. The reaction solution used for the preparation of Ag nanoparticles in the absence of Cl^- was also diluted at different ratios with preheated EG containing PVP-55k and Cl^-. The final concentrations of PVP and Cl^- in the diluted samples were

3.47 mg/mL and 0.21 mM, respectively, consistent with those of the reaction solution used for the synthesis of Ag nanocubes. An aliquot of 25 μL was withdrawn from the diluted sample for SERS measurement.

We also recorded SERS spectra from the reaction solution held at a temperature close to what was used for the synthesis. In this case, an aliquot of 25 μL was withdrawn from the hot reaction system as soon as the main LSPR peak reached 430 nm. The aliquot was transferred into the PDMS cell placed on a heating pad with its temperature preset to 150 ^{o}C. Thermal images were captured from the sample holder using an infrared camera to ensure that the temperature inside the PDMS cell was close to 150 ^{o}C. We then collected SERS spectra in the extended mode at an excitation wavelength of 532 nm, together with a 100× objective lens, a laser power at 25 mW, and a collection time of 10 s.

Instrumentation and Characterizations. We used a centrifuge (Eppendorf 5430) to collect and wash all solid products. A Cary 50 spectrometer (Agilent Technologies, Santa Clara, CA) was used to record the UV–vis spectra. Transmission electron microscopy (TEM) images were taken using a Hitachi HT7700 microscope (Japan) operated at 120 kV. The Raman and SERS spectra were recorded using a Renishaw inVia Raman Spectrometer (Wotton-under-Edge, U.K.) integrated with a Leica microscope (Wetzlar, Germany). Hydrodynamic diameters and zeta potentials were determined using a Zetasizer Nano ZS (Malvern Instruments, Worcestershire, UK).

2. Supplementary figures

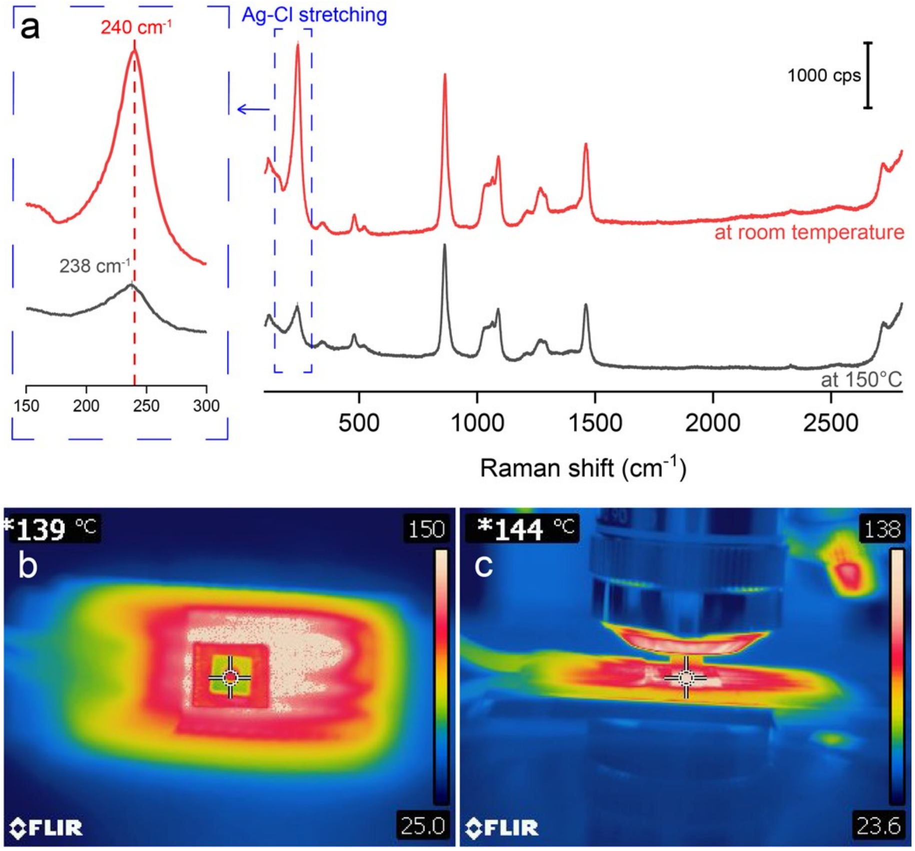

Figure S1. (a) SERS spectra recorded from the original reaction solution held at room temperature and about 150 ^{o}C, respectively. (b, c) Infrared thermal images recorded from the sample holder used to conduct SERS measurement, confirming that the sample indeed had at a temperature close to 150 ^{o}C.

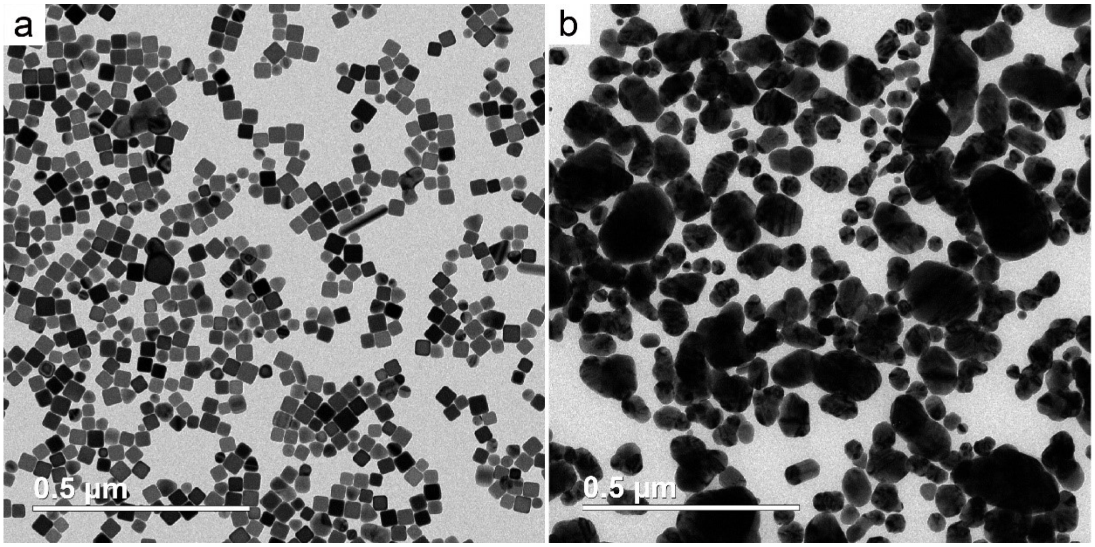

Figure S2. TEM images of the Ag (a) nanocubes and (b) irregular particles (prepared with CF_3COOAg in the absence of Cl^-), respectively, used for the measurement of hydrodynamic diameter.

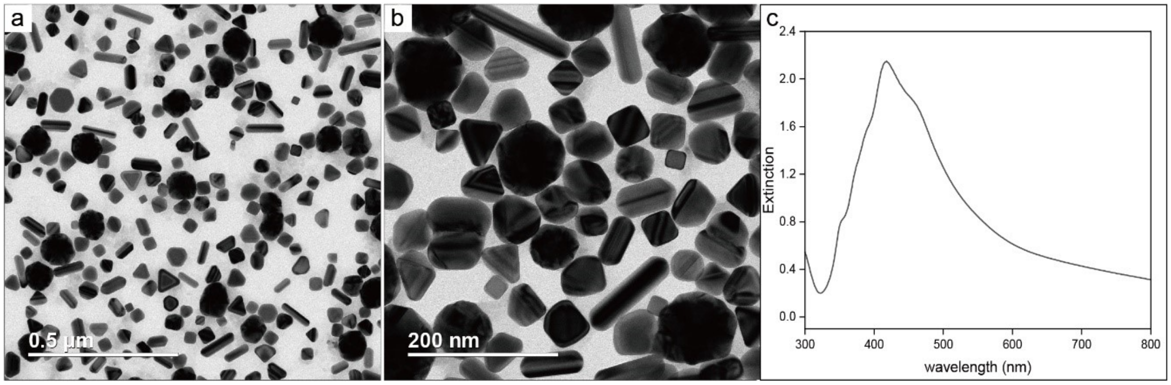

Figure S3. (a, b) TEM images of the Ag irregular particles prepared with $AgNO_3$ in the absence of Cl^-. The particles were collected by crushing with acetone, followed by centrifugation and washing with water. (c) UV-vis spectrum recorded from a suspension of the particles in EG.

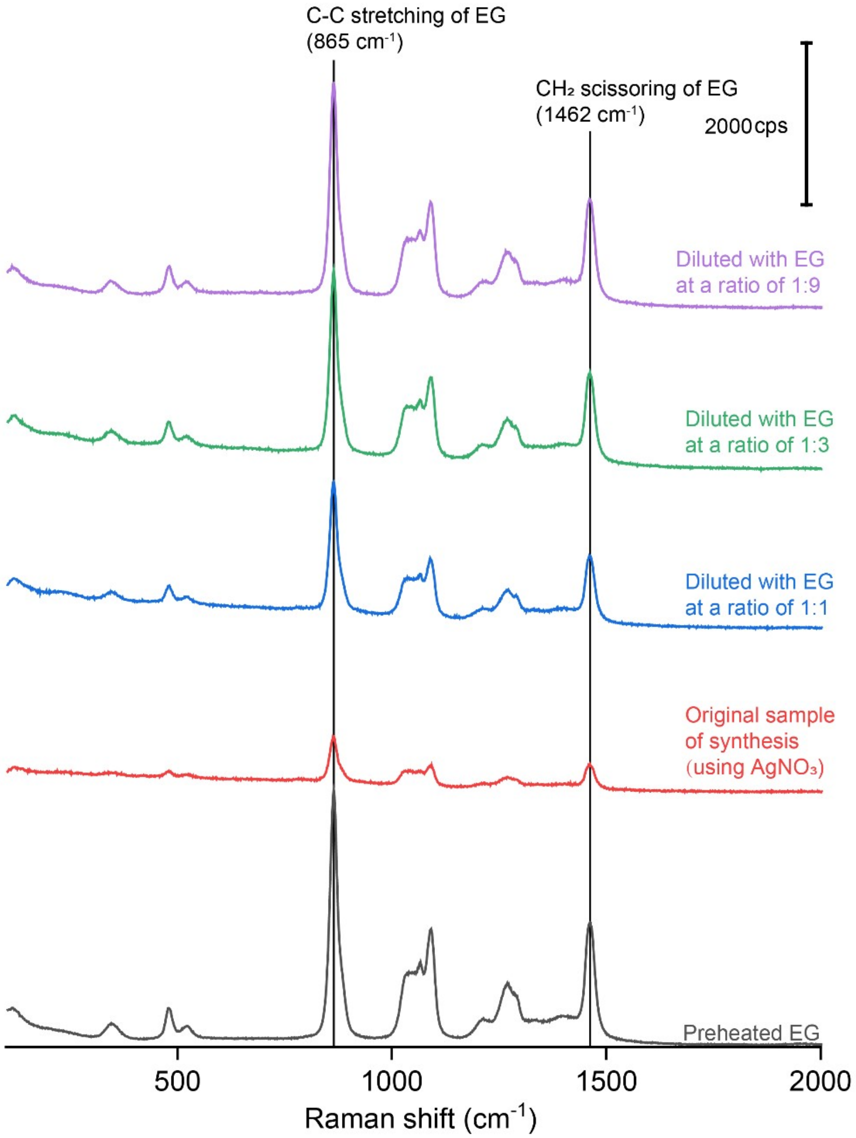

Figure S4. Raman spectrum of preheated EG and SERS spectra recorded from the original reaction solution of Ag irregular particles prepared with $AgNO_3$ in the absence of Cl^- and after it had been diluted with the EG at various ratios.

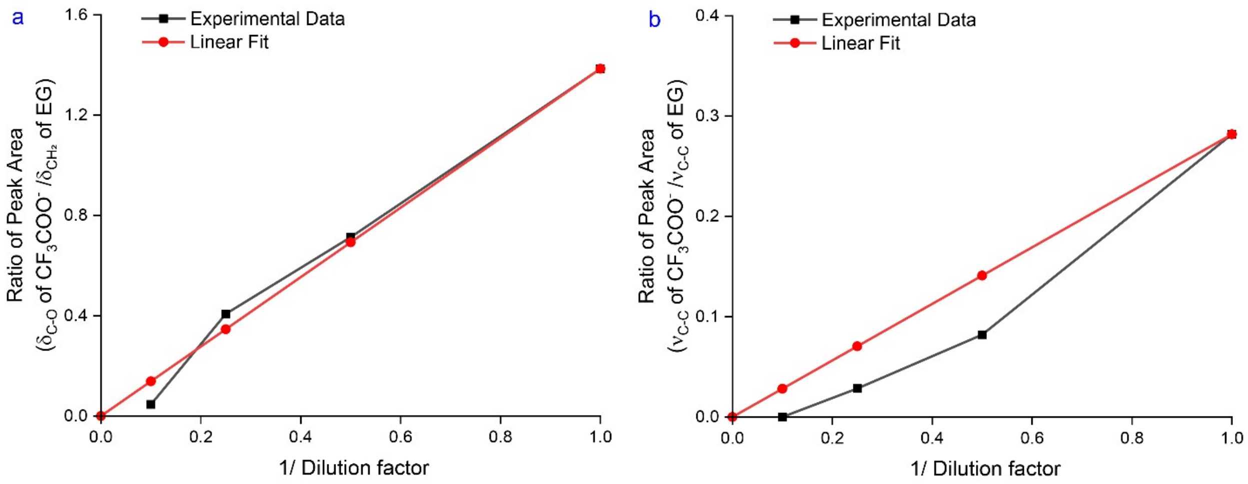

Figure S5. (a) Plots of the ratio between the areas of the peaks at 1435 cm^{-1} (deformation mode of C-O in CF_3COO^-) and 1462 cm^{-1} (scissoring mode of CH_2 in EG) as a function of the reciprocal of the dilution factor. (b) Plots of the ratio between the areas of the peaks at 847 cm^{-1} (ν_{C-C} of CF_3COO^-) and 865 cm^{-1} (ν_{C-C} of EG) as a function of the reciprocal of the dilution factor. The area of the peak was obtained by fitting the peak using a Gaussian–Lorentzian function.

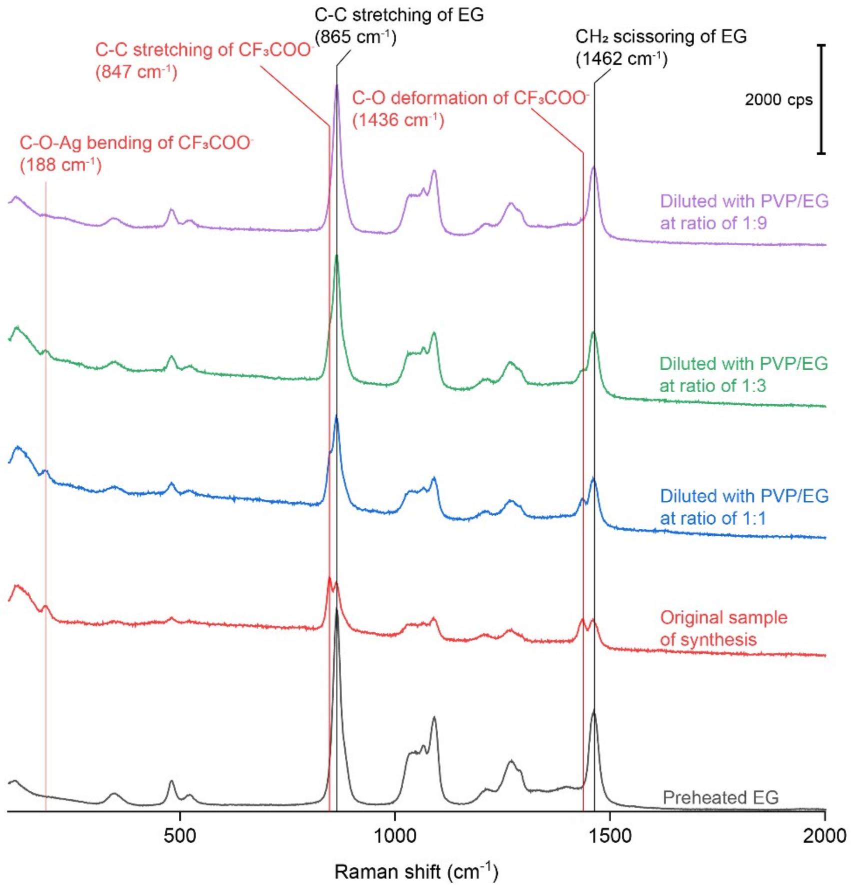

Figure S6. Raman spectrum of preheated EG and SERS spectra recorded from the original reaction solution and after it had been diluted with preheated EG containing PVP at various ratios, with the final concentration of PVP being 3.47 mg/mL.
